# Cost-effectiveness of mini-laparotomy in patients with colorectal cancers: A propensity scoring matching approach

**DOI:** 10.1371/journal.pone.0209970

**Published:** 2019-01-09

**Authors:** Herng-Chia Chiu, Hui-Min Hsieh, Chi-Lin Wan, Hsiang-Lin Tsai, Jaw-Yuan Wang

**Affiliations:** 1 Department of Healthcare Administration and Medical Informatics, Kaohsiung Medical University, Kaohsiung, Taiwan; 2 Research Education and Epidemiology Center, Changhua Christian Hospital, Changhua, Taiwan; 3 Institute of Hospital Management, Tsinghua University, China; 4 Department of Health Policy and Management, Bloomberg School of Public Health, John Hopkins University, Baltimore, Maryland, United States of America; 5 Department of Public Health, Kaohsiung Medical University, Kaohsiung, Taiwan; 6 Department of Medical Research, Kaohsiung Medical University Hospital, Kaohsiung, Taiwan; 7 Department of Community Medicine, Kaohsiung Medical University Hospital, Kaohsiung, Taiwan; 8 Division of General Surgery Medicine, Department of Surgery, Kaohsiung Medical University Hospital, Kaohsiung Medical University, Kaohsiung, Taiwan; 9 Department of Surgery, Faculty of Medicine, College of Medicine, Kaohsiung Medical University, Kaohsiung, Taiwan; 10 Division of Colorectal Surgery, Department of Surgery, Kaohsiung Medical University Hospital, Kaohsiung Medical University, Kaohsiung, Taiwan; 11 Graduate Institute of Clinical Medicine, College of Medicine, Kaohsiung Medical University, Kaohsiung, Taiwan; 12 Department of Surgery, Faculty of Medicine, College of Medicine, Kaohsiung Medical University, Kaohsiung, Taiwan; 13 Division of Colorectal Surgery, Department of Surgery, Kaohsiung Medical University Hospital, Kaohsiung Medical University, Kaohsiung, Taiwan; 14 Center for Biomarkers and Biotech Drugs, Kaohsiung Medical University, Kaohsiung, Taiwan; Universitat Bremen, GERMANY

## Abstract

**Objective:**

Surgical technique process innovations are expected to generally incur no additional cost but gain better quality. Whether a mini-laparotomy surgery (MLS) in the treatment of colorectal cancer (CRC) is more cost effective than conventional open surgery had not been well examined. The objective of this study was to apply cost-effectiveness approaches to investigate the cost effectiveness of adopting MLS compared with open surgery 1 year following resection in CRC patients.

**Research design:**

A prospective non-randomized cohort study design

**Setting:**

An academic medical center

**Participants:**

A total of 224 patients who received elective MLS and 339 who received conventional surgery; after propensity score matching, 212 pairs were included for analysis.

**Intervention:**

None

**Main outcome measures:**

Cost measures were hospital-index cost and outpatient and inpatient costs within 1 year after discharge. Effectiveness measures were life-years (LYs) gained and quality-adjusted life-year (QALYs) gained.

**Statistical methods:**

We calculated incremental costs and effectiveness by differences in these values between MLS and conventional surgery using adjusted predicted estimates.

**Results:**

MLS patients had lower rates of blood transfusions, less complication, and shorter post-surgical lengths of stay and more medical cost savings. One-year overall medical costs for MLS patients were TWD 748,269 (USD 24,942) per QALY gained, significant lower than for the comparison group (p-value = 0.045).

**Conclusion:**

Our findings supported that the less invasive surgical process through MLS not only saved medical costs, but also increased QALYs for surgical treatment in CRC patients.

## Introduction

Colorectal cancer (CRC) ranks as the third most common cancer in the US [1] and in European populations [2]. In Taiwan, CRC is the most common cancer and the third leading cancer cause of death [3]. Surgical management, such as open surgery and laparoscopic surgery, are two primary treatments for CRC in Taiwan [4]. Laparoscopically assisted resection for CRC patients has been further proved to an effective surgical methods in addition to conventional open surgery [5–7]. Currently, a mini-laparotomy surgical approach (MLS) is a new process innovation to CRC without laparoscopic assistance and is expected to reduce operative stress and promote early recovery of patients while maintaining the same curative resection rate as observed for conventional laparotomy [8–13].

MLS is defined as curative resection performed through a skin incision less than 8 cm in length [9, 13]. Mini-laparotomy surgery generally incurs no extra cost but achieved the same quality of care [14]. For example, existing studies showed that MLS, compared with conventional surgery, demonstrated shorter hospital stays and showed better short-term outcomes in the management of CRC patients [8–11, 13, 15]. Nevertheless, most studies focused on healthcare utilization and/or cost [16, 17], or clinical outcomes when comparing the outcomes of MLS with various surgical approaches [10, 11, 15, 18–21]. Very few studies have analyzed cost and effectiveness simultaneously by comparing medical costs and quality-adjusted life-years (QALYs) in CRC patients who underwent MLS procedures. The QALY-adjusted health economic evaluation models have been widely adopted in medical society to evaluate alternatives of healthcare treatments [22–25]. Whether the MLS approach achieved better cost effectiveness, as measured by incremental QALYs, had not been well examined.

The objective of this study was to apply cost-effectiveness methods to investigate the cost effectiveness of adopting MLS compared with conventional open surgery within 1 year following resection in CRC patients. Specifically, the first aim was to compare the short-term clinical outcomes between these two approaches; the second objective was to examine the differences in utility, QALYs, and cost, and further to investigate the incremental cost-effectiveness ratio (ICER) of the surgical process innovation.

## Methods

### Study design and data source

This study used a prospective cohort study design. Our study cohorts included patients who underwent CRC surgery at the Kaohsiung Medical University Hospital from 2007 to 2012. This study was approved by the Institutional Review Board (IRB) at Kaohsiung Medical University Hospital. Patients who received MLS surgery were classified as the process innovation group and those who received conventional open surgery were classified as the comparison group.

Study data were from several sources in order to conduct the cost-effectiveness analysis. First, to obtain patient health-related quality of care information, we interviewed patients using the European Organization for Research and Treatment Quality of life Questionnaire (EROTC QLQ-C30) quality instrument during the index hospitalization, and 3, 6, and 12 months post-discharge, to gather health-related quality of life data. The EROTC QLQ-C30 has been widely applied internationally in the literature to measure CRC patient quality of life [26–29]. The Chinese version of EROTC also demonstrated satisfactory reliability and validity [30–32]. Second, in order to compare the effectiveness of the types of surgeries, we extracted patient clinical information from electronic medical records, supplemented by written charts reviewed by experienced certified coders. The information included patient demographics and disease-related and treatment parameters. In addition, we obtained death information from the national registry of birth and death. The Taiwan death registry is an accurate and complete databases because it register all population death causes and the dates of death in Taiwan [33–35]. Finally, for cost measures, we extracted indexed hospitalization medical costs and follow-up outpatient and inpatient direct costs (including out-of-package and insurance coverage costs) from the electronic medical records in the study hospital.

### Study population

Patients who were newly diagnosed with primary CRC, American Joint Committee on Cancer (AJCC) stage I-III, and who received elective surgery during the patient identification period from 2007 to 2012 were potential study subjects. The MLS approach for the process innovation group was defined as curative resection performed through a skin incision less than or equal to 8 cm in length and were performed by using traditional surgical techniques and instruments. Patients were not eligible to receive MLS procedure and were excluded entirely based on the following criteria: 1) patients who did not consent to the procedure; 2) patients with tumors larger than 8 cm in size or with tumors that were infiltrating adjacent organs; 3) patients who had a previous abdominal operation; 4) patients who had either multiple carcinomas of the colon or adenomatous *polyposis coli*; 5) patients with severe intra-abdominal adhesions due to a prior laparotomy, 6) patients who had larger than the value of 28 in body mass index (BMI) [11, 13, 36], and 7) patients with acute total obstruction or perforation complications were also excluded. To avoid the potential selection bias, the above criteria were also applied upfront as exclusion criteria for the open surgery comparison group in the current study. Similar oncological principles were applied in both the conventional surgical and mini-laparotomy surgical approach groups of patients [13]. All patients received elective surgery and detailed studies included laboratory data analyses, colonofiberscopy, image studies (i.e., abdominal computed tomography; chest X-ray) [13]. Clinical stages and pathological features of the primary tumors were defined according to the seventh edition of the Union for International Cancer Control tumor–node–metastasis (TNM) staging system [13, 36, 37].

A total of 224 patients who received elective MLS and 339 patients who received conventional open surgery at our hospital were included and followed up for one year from the index date (discharge from hospital) or death. To eliminate the potential selection bias of the innovation group, in addition to the exclusion criteria described above, we further used propensity score matching (PSM) to match the compatible comparison group. Using a logistic regression model, we created propensity scores that predicted the probability of receiving the MLS procedure. The PSM caliper matching method with 1-to-1 match was used to match MLS patients with the conventional open surgery comparison group based on propensity score. The covariates included patient demographic characteristics (age and gender), cancer staging (AJCC 1-III), and Charlson Comorbidity Index (CCI). The CCI is a valid measure of disease severity [38]. The CCI was categorized as 0, 1, and ≥ 2.

### Outcomes metrics

#### Cost measures

Two direct medical costs were used, one the hospital-index cost and the other outpatient and inpatient costs within 1 year after discharge. Hospital index costs were sums of 16 cost objectives during the stay, including surgeon fees, medications, etc. One-year follow-up costs included costs for ambulatory cancer visits and costs associated with all-cause hospitalizations. We assumed that MLS patients would have lower hospital-index cost and 1-year follow-up cost due to better short-term clinical outcomes. Costs are presented in Taiwan dollar (TWD). The exchange rate between TWD and US dollars is about 30:1 in this study. All medical costs were adjusted to the 2012 consumer price index for medical goods.

#### Effectiveness measures

We measured clinical outcomes (e.g., blood transfusions, major and minor surgical complications rates, and length of hospital stay) and healthcare utilization during index hospitalization between MLS and conventional surgery patients. Types of complications were identified, including major surgical complications (e.g., anastomotic leakage, sepsis, abdominal abscess, respiration failure, pneumonia, bleeding, and other major complications) and minor surgical complications (e.g., urinary tract infection, intestinal obstruction, abdominal wound infection, gastrointestinal tract bleeding, and other minor complications) [39]. In addition, we calculated 1-year life-years (LYs) and quality-adjusted life-years (QALYs) as effectiveness measures given that innovative treatment may decrease risks of surgical complications or death and thus increase LYs. LYs were measured from the index hospital surgical date until death or the date of last follow-up within 1 year in the censored data. EROTC QLQ-C30, a multidimensional generic measure of health-related quality of life for cancer patients was used to measure patients’ perceived quality of care post-discharge. Following previously published preference-based algorithms, the EROTC QLQ-C30 scores were converted into utility weights, ranging between zero and one [40, 41]. The higher the utility weight, the better the health-related quality of life. QALYs were calculated by multiplying LYs by utility weight of each patient in both groups.

### Economic and statistical analytical approach

We conducted a cost-utility analysis to address our research questions. The time horizon was 1 year. Multiple generalized linear regressions were performed to control the variation, and a heteroskedasticity-robust standard error adjustment was used. We then calculated incremental costs and effectiveness by differences in these values between MLS and conventional surgery patients using adjusted predicted estimates. In addition, we calculated the ICER as the ratio of the incremental costs and divided by incremental effectiveness [14, 42]. All incremental measures were adjusted by patient demographic and clinical characteristics. Bootstrapping with 500 replications with sample size equivalent to the original was used to obtain standard errors for the incremental measures. Each point of bootstrapped estimate of the adjusted incremental effectiveness and costs was generated and then plotted in an incremental cost-effectiveness plane [14, 42, 43]. All statistical operations were performed using SPSS 20 (SPSS, Inc., Chicago, IL, USA) and Stata SE 12 version. A *P* value < 0.05 was considered significant.

## Results

Patient baseline demographic and disease-related characteristics were compared between the two groups before and after 1-to-1 PSM matching (Table 1). Before PSM matching, 224 MLS and 339 conventional surgery patients were included. Significant differences between the groups were found in the variables CCI (*P* = 0.002) and ileus on admission (*P* = 0.002). After PSM matching, all variables were similar between the two groups.

**Table 1:** Baseline characteristics before and after PSM matching, conventional surgery and mini laparotomy CRC patients between 2007 and 2012.

Variables		Before PSM Matching				P	After PSM Matching				P
		Conventional (N = 339)		Mini-laparotomy (N = 224)			Conventional (N = 212)		Mini-laparotomy (N = 212)		
		N/Mean	%/SD	N/Mean	%/SD		N/Mean	%/SD	N/Mean	%/SD	
**Demographic characteristics**											
Age (in years)		64.97	±12.90	65.16	±11.91	0.858	65.02	±13.07	65.05	±11.65	0.984
Age group*	< 65	160	47.2	99	44.2	0.728	96	45.3	95	44.8	0.945
	65–74	92	27.1	67	29.9		62	29.2	65	30.7	
	≧75	87	25.7	58	25.9		54	25.5	52	24.5	
Gender*	Male	189	55.8	128	57.1	0.811	120	56.6	119	56.1	1.000
	Female	150	44.2	96	42.9		92	43.4	93	43.9	
BMI*	< 18.5	32	9.4	12	5.4	0.203	23	10.8	12	5.7	0.136
	18.5–24	183	54.0	124	55.4		114	53.8	116	54.7	
	≧24	124	36.6	88	39.3		75	35.4	84	39.6	
**Disease-related characteristics**											
CCI*	0	126	37.2	86	38.4	0.002	91	42.9	84	39.6	0.458
	1–2	144	42.5	117	52.2		97	45.8	109	51.4	
	≧3	69	20.4	21	9.4		24	11.3	19	9.0	
Tumor location*	Colon	221	65.2	151	67.4	0.586	135	63.7	142	67.0	0.540
	Rectum	118	34.8	73	32.6		77	36.3	70	33.0	
Partial ileus on admission*	No	279	82.3	206	92.0	0.002	177	83.5	195	92.0	0.112
	Yes	60	17.7	18	8.0		35	16.5	17	8.0	
ASA group*	Ⅰ-Ⅱ	107	31.6	77	34.4	0.577	67	31.6	76	35.8	0.431
	Ⅲ-Ⅳ	227	67.0	145	64.7		143	67.5	135	63.7	
	Missing	5	1.5	2	0.9		2	0.9	1	0.5	
AJCC Stage*	I	66	19.5	51	22.8	0.364	44	20.8	50	23.6	0.780
	Ⅱ	106	31.3	76	33.9		73	34.4	71	33.5	
	Ⅲ	167	49.3	97	43.3		95	44.8	91	42.9	
Tumor grade level*	1	29	8.6	18	8.0	0.692	16	7.5	16	7.5	0.595
	2	275	81.1	189	84.4		172	81.1	179	84.4	
	3	32	9.4	17	7.6		23	10.8	17	8.0	
	Missing	3	0.9	0	0.0		1	0.5	0	0.0	
Lymphatic violations*	No	203	59.9	132	58.9	0.890	136	64.2	125	59.0	0.318
	Yes	136	40.1	92	41.1		76	35.8	87	41.0	
** Treatment-related characteristics**											
Operative time (min)*		202.64	±79.22	199.25	±68.94	0.631	202.55	±82.92	199.76	±67.87	0.725
Stoma*	No	248	73.2	178	79.5	0.108	155	73.1	168	79.2	0.171
	Yes	91	26.8	46	20.5		57	26.9	44	20.8	
Chemotherapy*	NO	137	40.4	79	35.3	0.254	89	42.0	74	34.9	0.162
	Yes	202	59.6	145	64.7		123	58.0	138	65.1	
Radiation therapy*	No	278	82.0	186	83.0	0.841	174	82.1	175	82.5	1.000
	Yes	61	18.0	38	17.0		38	17.9	37	17.5	
Timing of surgery	2007	87	25.7	25	11.2	<0.001	53	25.0	25	11.8	<0.001
	2008	60	17.7	37	16.5		34	16.0	32	15.1	
	2009	45	13.3	57	25.4		26	12.3	54	25.5	
	2010	69	20.4	61	27.2		49	23.1	60	28.3	
	2011	54	15.9	7	3.1		34	16.0	7	3.3	
	2012	24	7.1	37	16.5		16	7.5	34	16.0	

AJCC American Joint Committee on Cancer; BMI, body mass index; CCI: Charlson Comorbidity Index

* Variables with asterisks were included in the propensity score matching approach.

Table 2 presents the short-term clinical outcomes and index-hospital resource utilization between the two groups. MLS patients had lower rates of blood transfusion, less complication, and shorter post-surgical lengths of stay (all *P* < 0.05). For example, 32.1% of conventional surgery patients, compared with 22.6% of MLS patients, received blood transfusions during their hospital stays (*P* = 0.038). Conventional surgery patients stayed 2.38 days longer after the surgery (13.01 vs. 10.63 days; *P* = 0.009). One-year mortality rates were 11.8% for conventional surgery patients and 2.8% for MLS patients (*P* < 0.001).

**Table 2:** Short-term clinical outcomes and resources utilization, two surgical approaches.

Variables		Surgical Approach				*P*
		Conventional (N = 212)		Mini-laparotomy (N = 212)		
		N/Mean	%/SD	N/Mean	%/SD	
Blood transfusion	No	144	67.9	164	77.4	0.038
	Yes	68	32.1	48	22.6	
Blood transfusion(*volume)*		324.64	±966.97	197.87	±632.65	0.112
Complications^a^	No	185	87.3	196	92.5	0.108
	Yes	27	12.7	16	7.5	
Admitted to ICU	No	195	92.0	198	93.4	0.709
	Yes	17	8.0	14	6.6	
Index ALOS		18.34	±13.65	15.58	±5.41	0.007
Post-surgical ALOS		13.01	±12.33	10.63	±4.54	0.009
One year survival	Survive	187	88.2	206	97.2	0.001
	Death	25	11.8	6	2.8	

ALOS, average length of stay; ICU, intensive care unit.

^a^ Types of complications included major surgical complications (e.g., anastomotic leakage, sepsis, abdominal abscess, respiration failure, pneumonia, bleeding and other major complications) and minor surgical complications (e.g., urinary tract infection, intestinal obstruction, abdominal wound infection, gastrointestinal tract bleeding and other minor complications). Counts for the surgical complications were listed in S1 Table.

Table 3 lists 16 items of direct medical costs during index hospitalizations between conventional surgery and MLS. CRC patients who underwent MLS tended to have lower direct medical costs. For example, the overall medical costs for the index hospitalization were TWD 169,701(USD 5,656) for conventional surgery and TWD 134,537(USD 4,484) for MLS; special material costs (e.g. auto suture, needle, cannula, or wound nursing) were TWD 10,787(USD 360) for conventional surgery and TWD 8,713(USD 290) for MLS; and surgical costs were TWD$46,614(USD 1,550) for conventional surgery and TWD 44,622(USD 1,487) for MLS.

**Table 3:** Index-hospitalization direct medical Costs between CRC patients who underwent two surgical approaches.

Cost item	Total (N = 424)							Conventional (N = 212)							Mini-laparotomy (N = 212)						
	Mean	±	SD	Median	Quartile			Mean	±	SD	Median	Quartile			Mean	±	SD	Median	Quartile		
					25	50	75					25	50	75					25	50	75
Diagnosis costs	6,347	±	4,320	5,391	4,282	5,391	6,913	6,977	±	5,757	5,430	4,213	5,430	7,794	5,717	±	1,864	5,374	4,514	5,374	6,645
Ward costs	24,685	±	26,335	19,260	14,726	19,260	26,432	28,629	±	35,870	19,699	14,620	19,699	27,685	20,742	±	8,512	18,320	14,726	18,320	24,812
Laboratory costs	14,848	±	9,155	12,734	9,056	12,734	17,791	16,114	±	11,448	12,879	9,102	12,879	19,245	13,581	±	5,811	12,490	8,936	12,490	17,099
X-Ray costs	5,304	±	4,205	5,601	770	5,601	6,340	5,714	±	4,930	5,601	631	5,601	6,513	4,894	±	3,288	5,598	834	5,598	6,155
Therapeutic procedure costs	7,688	±	12,944	4,881	3,520	4,881	8,224	9,595	±	17,851	5,166	3,482	5,166	8,715	5,781	±	3,153	4,748	3,578	4,748	7,322
Surgical costs	45,618	±	9,570	44,556	40,556	44,556	49,433	46,614	±	11,681	44,556	40,556	44,556	51,746	44,622	±	6,720	44,119	40,556	44,119	48,508
Anesthesia costs	13,310	±	5,212	11,809	10,034	11,809	15,681	13,783	±	6,277	11,696	9,325	11,696	16,905	12,837	±	3,821	11,809	10,230	11,809	15,116
Special materials costs	9,750	±	9,052	7,826	4,717	7,826	11,934	10,787	±	11,955	8,188	3,798	8,188	12,914	8,713	±	4,379	7,675	6,033	7,675	11,478
Drug costs	16,565	±	32,008	9,032	5,367	9,032	14,926	20,751	±	43,401	8,484	4,762	8,484	16,602	12,379	±	11,621	9,510	6,108	9,510	14,298
Dispensing service costs	2,632	±	2,256	2,140	1,125	2,140	3,436	2,720	±	2,743	1,715	1,079	1,715	3,415	2,544	±	1,631	2,524	1,168	2,524	3,438
Injection service costs	1,580	±	1,665	1,375	386	1,375	2,103	1,619	±	2,042	989	312	989	2,103	1,541	±	1,177	1,591	460	1,591	2,109
Other costs^a^	3,792	±	15,910	0	0	0	1,648	6,396	±	21,916	909	0	909	2,404	1,188	±	3,679	0	0	0	1,205
**Total**	152,119	±	110,496	130,007	107,794	130,007	162,551	169,701	±	150,584	130,537	104,687	130,537	176,533	134,537	±	34,347	129,525	110,041	129,525	152,948

^a^ Other costs included Tube feeding costs, rehabilitation costs, blood/plasma costs, hemodialysis costs, and psychiatric treatment costs

Table 4 presents the incremental estimates and ICERs by medical costs and QALYs for the MLS and conventional surgery groups. MLS patients showed statistically significant health outcome (LYs and QALYs) gains and cost savings compared with conventional surgery patients. Specifically, with regard to effectiveness of care, LYs for conventional surgery and MLS patients were 0.942 and 0.993. After multiplying the utility weight by LYs, those values, reinterpreted as QALYs, were 0.840 and 0.913, respectively. Adjusted incremental values were 0.037 (*P* < 0.01) per LYs gained and 0.060 (*P* < 0.001) per QALYs gained. With regard to cost savings of surgical process innovation, after adjusting for all covariates, the MLS approach saved TWD -20,417 (USD -680) (*P* < 0.05) in index-hospitalization medial costs, and TWD -45,224 (USD -1,507) in overall medical costs within 1 year (*P* < 0.05).

**Table 4:** Results for incremental effectiveness, medical costs and ICER between CRC patients who underwent two surgical approaches.

	Overall (N = 424) Mean (SD)			Conventional (N = 212) Mean (SD)			Mini-laparotomy (N = 212) Mean (SD)			Mini-laparotomy—Conventional			
										Unadjusted Increments^c^		Adjusted Increments^a^^,^^c^	
										(Bootstrap SE)		(Bootstrap SE)	
**Incremental Effectiveness**													
Utility score	0.906	±	0.055	0.891	±	0.05	0.920	±	0.06	0.028	(0.01)***	0.028	(0.01)***
Life-years	0.967	±	0.148	0.942	±	0.20	0.993	±	0.06	0.051	(0.01)***	0.037	(0.01)**
QALYs^1^	0.876	±	0.145	0.840	±	0.18	0.913	±	0.08	0.073	(0.01)***	0.060	(0.01)***
**Incremental direct medical costs**													
Index-hospitalization medical costs	152,119	±	110,496	169,701	±	150,584	134,537	±	34,347	-35,164	(9,964)***	-20,417	(8,776)*
Direct medical costs within 1 year follow-up period													
1-year outpatient medical costs	62,210	±	66,259	61,473	±	72,628	62,947	±	59,375	1,474	(6,047)	-2,520	(6,031)
1-year inpatient medical costs^b^	279,196	±	238,819	306,429	±	270,795	251,964	±	198,796	-54,465	(21,950)*	-42,704	(21,197)*
1-year overall medical costs (including outpatient and inpatient costs)	341,406	±	255,516	367,902	±	286,705	314,910	±	217,431	-52,991	(23,594)*	-45,224	(21,184)*
**Incremental Cost-Effectiveness Ratio (ICER)**													
1-year medical costs per LY gains													
Outpatient medical costs per LY gains										29,173	(127,443)	-67,232	(189,860)
Inpatient medical costs per LY gains										-1,078,153	(537,258)*	-1,139,419	(760,037)
Overall medical costs per LY gains										-1,048,980	(570,363)	-1,206,651	(773,774)
1-year medical costs per QALY gains													
Outpatient medical costs per QALY gains										20,295	(86,176)	-41,692	(106,018)
Inpatient medical costs per QALY gains										-750,070	(326,235)*	-706,577	(371,789)
Overall medical costs per QALY gains										-729,774	(351,138)*	-748,269	(372,592) *

QALY, quality-adjusted life-years

^a^ Models are adjusted for confounding variables listed in the Table 1, including gender, age, BMI, tumor location, CCI, ileus on admission, intestinal perforation or peritonitis, ASA, AJCC stage, tumor grade, lymphatic violations, stoma, chemotherapy, and radiation therapy.

^b^ Direct inpatient costs included medical and surgical expenditure within 1 year of index hospitalization.

^c^ * p<0.05, **p<0.01, ***p<0.001

**Fig 1:**
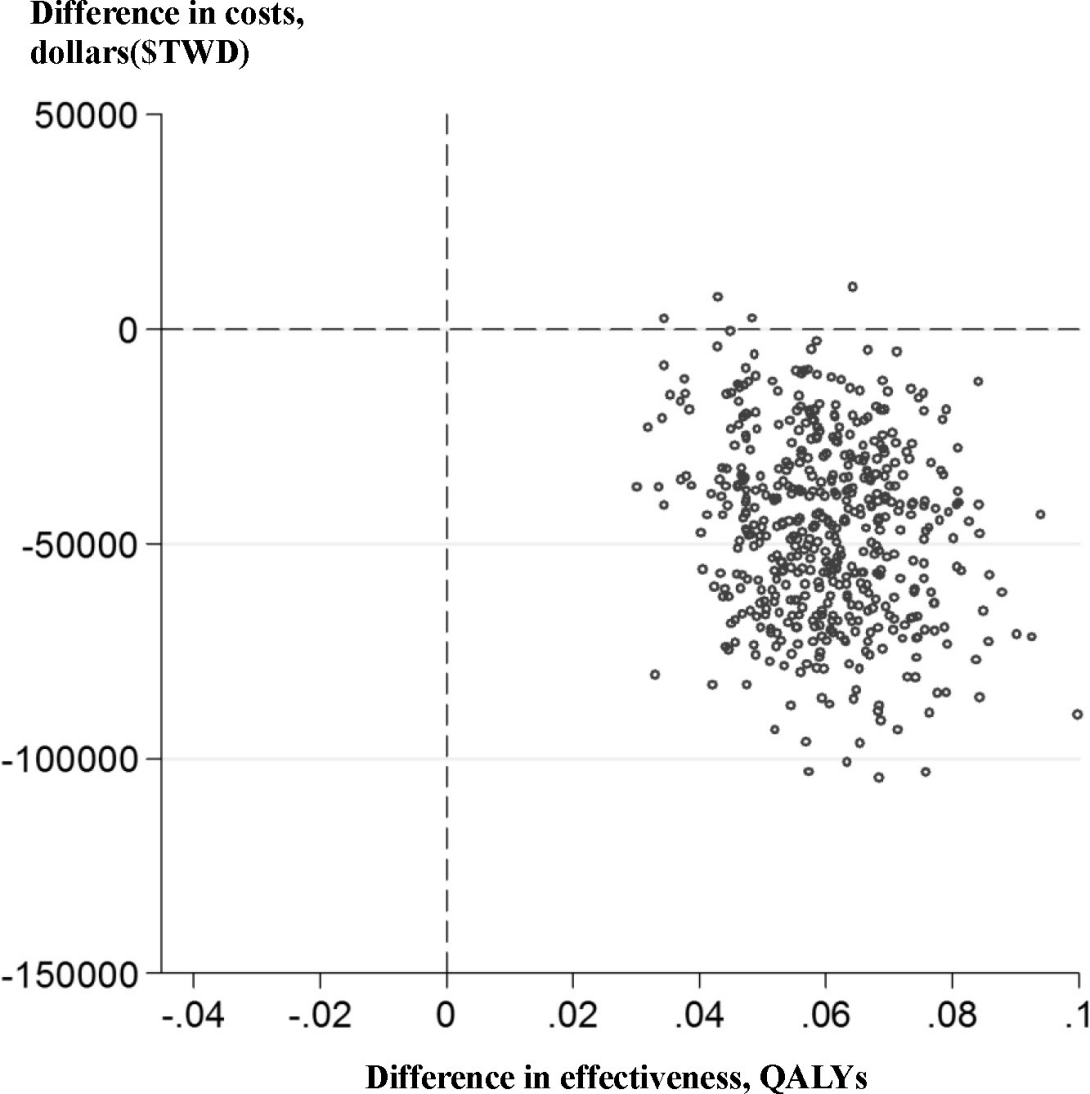
Incremental QALYs and incremental total costs between conventional and mini-laparotomy: 1 year after surgery.

Table 4 also shows that the ICERs for inpatient total cost by gains in LYs and QALYs for MLS patients were statistically greater than for conventional surgery patients in unadjusted increments. After adjustment for all covariates, 1-year overall medical costs per QALY gained was less in MLS patients (TWD 748,269 or USD 24,942) per QALY gained; *P* = 0.045) than for conventional open surgery patients. Fig 1 shows scatter plots for the distribution of incremental QALYs and incremental costs on the cost-effectiveness planes. MLS patients had lower 1-year overall medical costs and greater QALYs than conventional surgery patients (*P* = 0.001).

## Discussion

This study examined the cost effectiveness of MLS compared with conventional open surgery in the management of CRC patients undergoing radical resection. MLS resulted in decreased costs and increased QALYs, and appeared to offer great advantages (less costly, more effective). In particular, MLS offered better short-term clinical outcomes in index hospitalization measures, and greater QALY gains and dollars saved within 1 year after surgery.

With respect to the perioperative and short-term outcomes of the study, MLS resulted in lower rates of blood transfusion, fewer complications, shorter lengths of stay, and lower rate of mortality, compared with conventional surgery. These results were consistent with previous studies regarding the immediate advantage of innovated surgical procedures [8–11, 13, 15]. The post-surgical length of hospital stay is a better indicator of CRC surgical outcomes [39]. Our study indicated that MLS patients stayed 2 days fewer after the surgery than conventional open surgery patients (10.63 vs. 13.01 days), possibly due to less pains or complications after surgery. From a utilization management perspective, MLS not only decreased the index hospitalization stay, it also increased efficient use of hospital beds due to a higher turnover rate.

In addition, previous studies suggested that the laparoscopic surgery, as compared with convention surgery, demonstrated shorter hospital length of stay, but similar in overall mortality rates and perioperative complications [44, 45]. Our study had found that crude rate of 1-year mortality rate was much lower in the MLS group than the conventional surgical approach group. We further checked patients who died for their mean survival time within one year between conventional and MLS approach were 0.51 (n = 25, 11.8% of 212 matched CS group) and 0.74 years (n = 6, 2.8% of 212 matched MLS group), respectively; overall 52% of CS death and 33% of MLS death died within 180 days; 84% of CS death and 100% of MLS death due to CRC cause of death based on ICD-9-CM code 153 and 154. Several empirical evidences also suggested that mini-laparotomy seems a feasible, minimally invasive and safe alternatives to conventional laparotomy for Stage I-III CRC resection [11, 13, 36]. However, given the limitation of the current observational study using small sample size and the short-term follow-up period within one-year, caution is needed when interpreted the observed the potential benefit of MLS in reducing mortality between matched CS and MLS cohorts.

For cost-effectiveness analysis, the differences in effectiveness and costs between the two groups were statistically significant for all effectiveness and cost measures at both unadjusted and adjusted increments, with the exception of total outpatient cost. After covariate adjustment, compared with conventional surgery patients, MLS patients saved TWD 20,417 (USD 680) in index-hospital costs and TWD 45,224 (USD 1,507) in 1-year costs post-discharge. We further examined 16 cost items from the hospital stay and found that conventional surgery consumed more dollars for each cost item than MLS. Regarding effectiveness of care, after adjusting for all covariates, incremental values were 0.037 (*P* < 0.01) per LYs gained and 0.060 (*P* < 0.001) per QALYs gained for MLS patients. This indicates that patients receiving MLS have better health-related quality of life after discharge, compared with conventional surgery patients. Our study not only indicated different mortality rates between groups, but further investigate the utility score, LYs and QALYs between groups. The cost-effectiveness results provide addition information on the effectiveness of quality of care over time. Based on our study, we suggest that better outcomes in index-hospitalization (e.g., lower transfusion rates, lower complication rates), as a result of MLS, had greater impact on short-term clinical and financial outcomes for CRC patients.

Our study indicated that MLS used fewer hospital resources, saved healthcare dollars, and provided gains in quality of life. Recent studies have examined the benefits of laparoscopic surgery and robotic-assisted surgery compared with each other or with conventional surgery, and found that medical technological improvements improved surgical outcomes [12, 18–21, 46]. The new medical technology is believed to be driving improved healthcare; the only concerns relate to high unit costs for the services and aggregated costs for healthcare care expenditure. For instance, Kim and associates (2015) found that the cost of robotic surgery is statistically higher than the cost of laparoscopy surgery for rectal cancer patients [47]. That study concluded that these two surgical approaches are comparable for short- and long-term care outcomes, but higher costs may deserve attention given that national economics are constrained in many countries. In reality, many healthcare systems around the world are not as rich as in developed nations; their healthcare budgets are relatively limited. When the technological innovation approach is financially not feasible for the healthcare systems, the surgical process innovation is preferred to conventional surgery.

The current study has several limitations. First, the data we used were obtained from a single medical center in Taiwan, so the results may not generalize to other populations or samples. Second, given the study is lack of randomization and the potential confounders might be still existed. However, we used propensity scoring matching technique to find comparable innovation and comparison groups for minimizing the selection bias, and further to apply multiple variable regression models to control for the potential confounders. Third, the cost data were derived from one institutional health information system; costs incurred from other medical institutions were not available. Therefore, this study can only assume that CRC study patients in Taiwan tend to receive post-surgical care from the same institutions or physicians.

## Conclusions and implication

Cost-effectiveness analysis is an economic evaluation model widely used to identify advantages of substitute or alternative medical care in considering cost and effectiveness simultaneously. Based on this study, we confirmed that the less invasive surgical process not only saved medical cost, but also increased QALYs in CRC patients. We conclude that the MLS patients had shorter hospital stays and lower medical expenses in both the index hospitalization and total costs 1 year after discharge, in comparison to convention surgery patients. Moreover, MLS patients, compared with conventional surgery patients, gained more LYs, as well as QALYs. The less costly and more effective surgical method, mini-laparotomy, deserves attention.

## Supporting information

S1 Table Number of complications, conventional surgery and mini-laparotomy. (DOCX)

S1 File Minimal anonymized dataset_R2. (XLSX)

S2 File Revised-CHEERS-Checklist_R3. (PDF)
