# Supplementary material for: Cost-effectiveness of mini-laparotomy in patients with colorectal cancers: A propensity scoring matching approach
Source: PLoS One. 2019 Jan 9;14(1):e0209970. doi: 10.1371/journal.pone.0209970 (PMC6326502; doi:10.1371/journal.pone.0209970)
Supplement: S1 Table — (DOCX) [file pone.0209970.s001.docx]

S1 Table: Number of complications, conventional surgery and mini-laparotomy

| Complications | Surgical Approach | |
| --- | --- | --- |
|  | Conventional | Mini-laparotomy |
| **Major** |  |  |
| Anastomotic leakage | 2 | 0 |
| Sepsis | 7 | 4 |
| Abdominal abscess | 3 | 2 |
| Respiration failure | 3 | 0 |
| Pneumonia | 4 | 0 |
| Bleeding | 0 | 1 |
| Other major complication | 10 | 2 |
| **Minor** |  |  |
| Urinary tract infection | 4 | 3 |
| Intestinal obstruction | 3 | 1 |
| Abdominal wound infection | 2 | 0 |
| Gastrointestinal tract bleeding | 7 | 5 |
| Other minor complication | 7 | 3 |
